# Does Bacteriophage ϕ29 Pack Its DNA with a Twist?

**DOI:** 10.1371/journal.pbio.0050091

**Published:** 2007-02-20

**Authors:** Mary Hoff

You probably never tried to put toothpaste back into the tube, but if you did, you’d have a good idea of what the *Bacillus subtilis* bacteriophage ϕ29 experiences as it crams its DNA into a protein capsid shell following replication. Scientists have speculated that this bacteria-infecting “phage” accomplishes this energy-intensive task by rotating a connector complex at the opening that feeds the DNA into the capsid as it turns, and that this process is fueled by the breakdown of ATP by an associated ring of ATPases. But until now there has been no way to show whether that’s really what happens.

Thorsten Hugel, Jens Michaelis, Craig Hetherington, Carlos Bustamante, and colleagues set out to determine whether rotation is part of the packaging routine; they used single-molecule fluorescence polarization spectroscopy and single-molecule force spectroscopy—visualization tools described in more detail below.

The researchers started by developing a system for lining up capsids so they could be observed en masse while they package DNA. Their innovative system combined single-molecule fluorescence polarization with “magnetic tweezers,” which involves gluing the end of the capsid farthest from the hole to a slide using antibodies, then drawing out the DNA being packaged in the opposite direction by attaching a magnetic bead to its loose end and applying a magnetic field.

A second major preparatory task was to figure out a way to label the connector complex so its motion could be observed—the researchers did this by attaching fluorescent dye molecules to the connectors. Observations confirmed that neither the use of magnetic tweezers nor the attachment of dye molecules substantially interfered with the packaging process. Using single-molecule fluorescence polarization spectroscopy, the researchers could measure polarization from the dye-labeled connectors. By rotating the excitation polarization, they also showed that the setup had the characteristics necessary to yield useful information, and that it would be able to detect rotation rates of 0.2 to 5 rotations per second—the range needed to test for rotating DNA-loading mechanisms that had previously been proposed.

**Figure:**
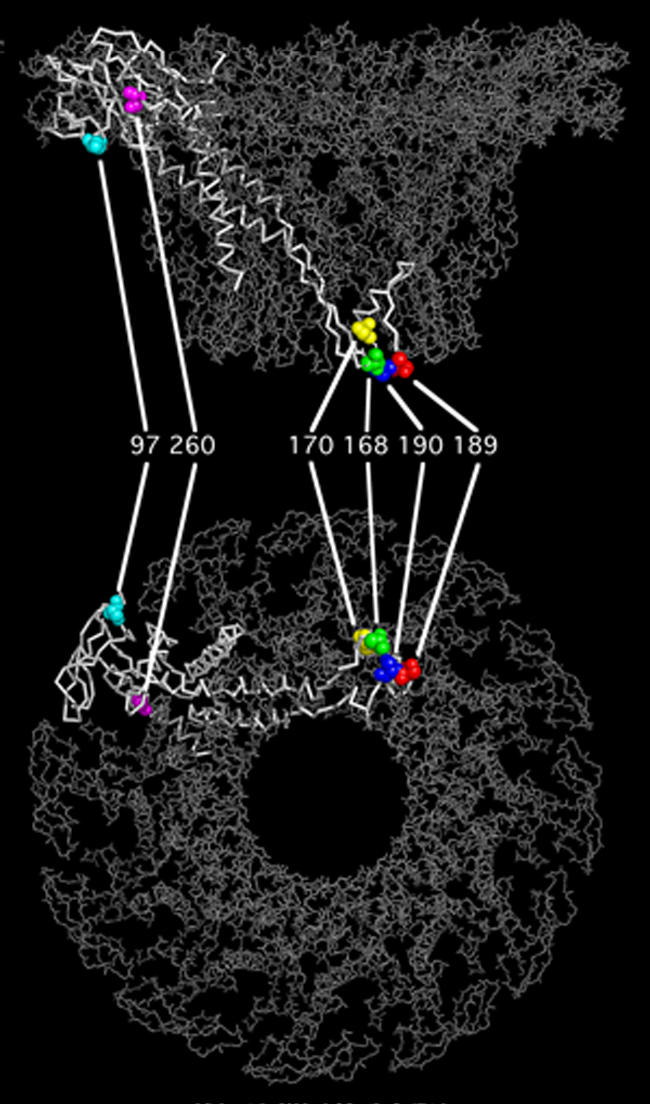
The bacteriophage ϕ29 DNA-packaging machine. Double-stranded DNA is driven into the preformed capsid shell by a complex and powerful molecular motor. Image: Precision Graphics (Champaign, Illinois), K. Aathavan and Y. Chemla (University of California Berkeley)

With their innovative experimental apparatus in place, the researchers proceeded to take a close-up look at connector movement during DNA packaging in six ϕ29 mutants. After attaching the fluorescent molecules to the connector complex, they set the mutants to work packaging DNA in a flow chamber. As the connectors functioned, the researchers shone homogeneously polarized light on them and recorded the pattern of fluorescence produced in two channels at right angles to one other.

If the connectors had been rotating, the researchers would have seen a sine-wave-like fluctuation in intensity in both channels with a phase shift of 90 degrees. Not only did they not see the sine waves, but mathematical analysis of the fluorescence pattern confirmed that the changes in the fluorescence emitted by the molecules as packaging took place did not correspond to any sort of continuous rotational motion. The researchers concluded with more than 99% certainty that the packaging mechanism does not involve rotation.

How, then, does it happen? The researchers noted that their findings are compatible with a recently proposed nonrotating model in which the ring of ATPases alternately compresses and extends, drawing the DNA in—a bit like what your mouth might do if you had to eat a plateful of spaghetti with your hands tied behind your back. But further testing will be needed to confirm the validity of that model to the degree of certainty with which this team rejected the rotator hypothesis.

