# Correction: Does Bacteriophage φ29 Pack Its DNA with a Twist?

**DOI:** 10.1371/journal.pbio.0050130

**Published:** 2007-05-15

**Authors:** Mary Hoff

In *PLoS Biology*, volume 5, issue 3: doi: 10.1371/journal.pbio.0050091

The incorrect image was published with this synopsis. The correct image, along with the caption, appears below.

## 

**Figure:**
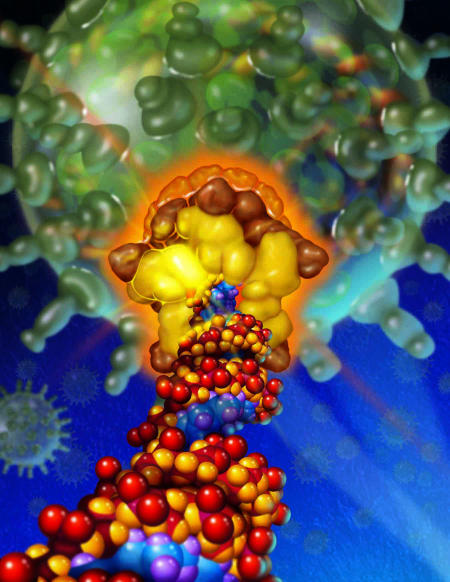
The bacteriophage φ29 DNA-packaging machine. Double-stranded DNA is driven into the preformed capsid shell by a complex and powerful molecular motor. Image: Precision Graphics (Champaign, Illinois), K. Aathavan and Y. Chemla (University of California Berkeley)

